# 1,8-Dibenzoyl­naph­tha­lene-2,7-diyl dibenzoate

**DOI:** 10.1107/S1600536812030991

**Published:** 2012-07-14

**Authors:** Rei Sakamoto, Kosuke Sasagawa, Daichi Hijikata, Akiko Okamoto, Noriyuki Yonezawa

**Affiliations:** a Department of Organic and Polymer Materials Chemistry, Tokyo University of Agriculture and Technology, 2-24-16 Naka-machi, Koganei, Tokyo, Japan

## Abstract

In the title compound, C_38_H_24_O_6_, the phenyl rings of the benzoyl and benzo­yloxy groups make dihedral angles of 67.12 (5), 85.15 (5), 76.41 (5) and 71.47 (5)° with the naphthal­ene ring system. In the crystal, C—H⋯O hydrogen bonds link mol­ecules into chains parallel to the *b* axis. The structure also features C—H⋯π and π–π stacking inter­actions, with centroid–centroid distances in the range 3.6441 (7)–3.9197 (8) Å.

## Related literature

For electrophilic aromatic aroylation of the naphthalene core, see: Okamoto & Yonezawa (2009 ▶); Okamoto *et al.* (2011 ▶). For the structures of closely related compounds, see: Mitsui *et al.* (2008 ▶); Nakaema, Imaizumi *et al.* (2008 ▶); Nakaema, Watanabe *et al.* (2008 ▶); Mitsui *et al.* (2008 ▶, 2009 ▶).
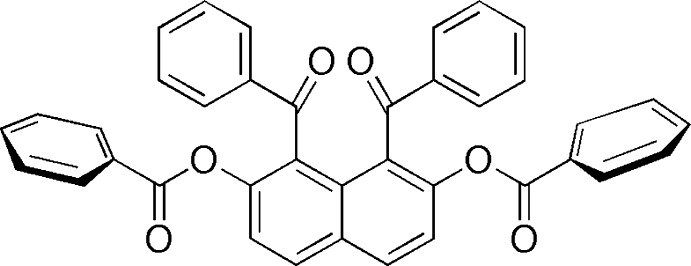


## Experimental

### 

#### Crystal data

C_38_H_24_O_6_
*M* *_r_* = 576.57
Orthorhombic, 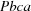

*a* = 18.0080 (3) Å
*b* = 12.4307 (2) Å
*c* = 25.3332 (4) Å
*V* = 5670.89 (16) Å^3^
*Z* = 8
Cu *K*α radiation
μ = 0.74 mm^−1^
*T* = 193 K
0.40 × 0.40 × 0.10 mm

#### Data collection

Rigaku R-AXIS RAPID diffractometer
Absorption correction: numerical (*NUMABS*; Higashi, 1999 ▶) *T*_min_ = 0.756, *T*_max_ = 0.930
95847 measured reflections
5182 independent reflections
4687 reflections with *I* > 2σ(*I*)
*R*_int_ = 0.023

#### Refinement

*R*[*F* ^2^ > 2σ(*F* ^2^)] = 0.032
*wR*(*F* ^2^) = 0.084
*S* = 1.05
5182 reflections
398 parameters
H-atom parameters constrained
Δρ_max_ = 0.18 e Å^−3^
Δρ_min_ = −0.16 e Å^−3^

### 

Data collection: *PROCESS-AUTO* (Rigaku, 1998 ▶); cell refinement: *PROCESS-AUTO*; data reduction: *CrystalStructure* (Rigaku, 2010 ▶); program(s) used to solve structure: *SIR2004* (Burla *et al.*, 2005 ▶); program(s) used to refine structure: *SHELXL97* (Sheldrick, 2008 ▶); molecular graphics: *ORTEPIII* (Burnett & Johnson, 1996 ▶); software used to prepare material for publication: *SHELXL97*.

## Supplementary Material

Crystal structure: contains datablock(s) I, global. DOI: 10.1107/S1600536812030991/rz2784sup1.cif

Structure factors: contains datablock(s) I. DOI: 10.1107/S1600536812030991/rz2784Isup2.hkl

Supplementary material file. DOI: 10.1107/S1600536812030991/rz2784Isup3.cml

Additional supplementary materials:  crystallographic information; 3D view; checkCIF report

## Figures and Tables

**Table 1:** Hydrogen-bond geometry (Å, °) *Cg*2 is the centroid of the C5–C10 ring.

*D*—H⋯*A*	*D*—H	H⋯*A*	*D*⋯*A*	*D*—H⋯*A*
C15—H15⋯O1^i^	0.95	2.41	3.0584 (17)	125
C28—H28⋯*Cg*2^i^	0.95	2.65	3.4877 (14)	148

Symmetry code: (i) 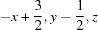.

**Fig. 1.**
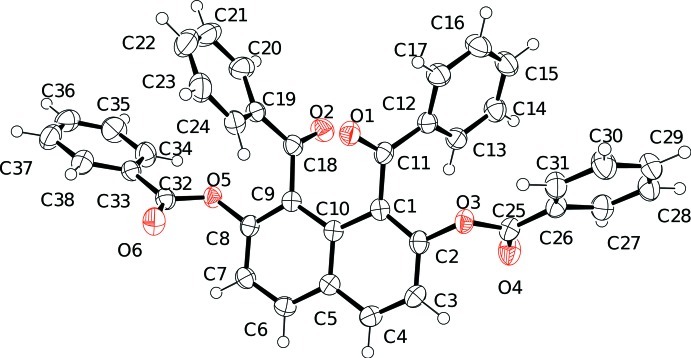
The molecular structure of title compound, showing 50% probability displacement ellipsoids.

**Fig. 2.**
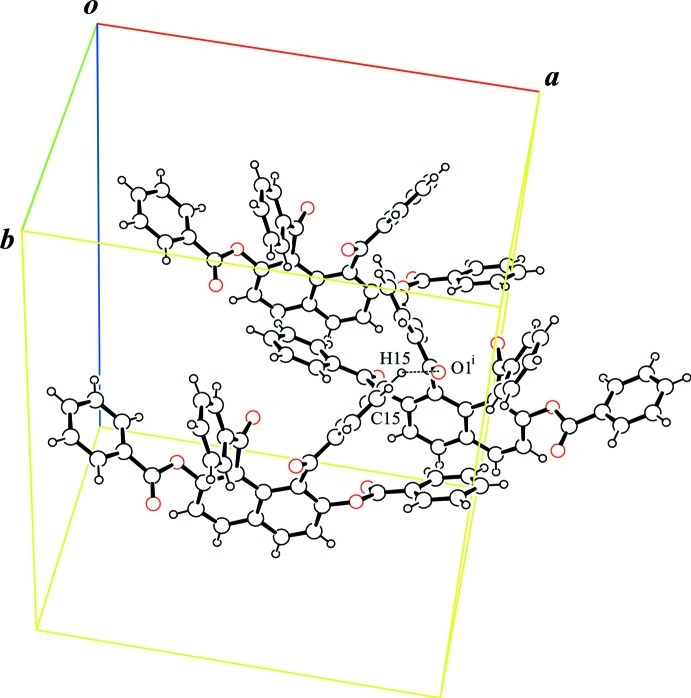
A partial crystal packing diagram of the title compound showing the C–H···O hydrogen interaction (dashed line). Symmetry code: (i) 3/2-x, -1/2+y, z.

## supplementary crystallographic information

### Comment

In the course of our study on electrophilic aromatic aroylation of the naphthalene core, 1,8-diaroylnaphthalene compounds have proved to be formed regioselectively by the aid of a suitable acidic mediator (Okamoto & Yonezawa, 2009, Okamoto *et al.*, 2011). Recently, we have reported the X-ray crystal structures of 1,8-diaroylnaphthalenes, *e.g.*, 1,8-dibenzoyl-2,7-dimethoxynaphthalene (Nakaema, Watanabe *et al.*, 2008). The aroyl groups at 1,8-positions of the naphthalene rings in these compounds are oriented in opposite directions. Furthermore, we have also investigated modification of 2,7-positions in 1,8-diaroylnaphthalene compounds and clarified the X-ray crystal structures of the resulting molecules such as (4-chlorophenyl)(2-hydroxy-7-methoxynaphthalene-1-yl)methanone (Mitsui *et al.*, 2008) and (4-chlorobenzoyl)(2-hydroxy-7-ethoxynaphthalene-1-yl)methanone (Mitsui *et al.*, 2009). Besides, the homologous aroyl group-free naphthalene derivative, 2,7-bis(4-acetylphenoxy)napthalene (Nakaema, Imaizumi *et al.*, 2008) has been revealed. As a part of our ongoing studies on the formation and crystal structure analyses of aroylated naphthalene derivatives, the crystal structure analysis of the title compound, 1,8-dibenzoylnaphthalene bearing benzoyloxy groups at the 2,7-positions, is discussed in this article.

The molecular structure of the title compound is displayed in Fig 1. The benzene rings of benzoyl groups and benzene rings of benzoyloxy groups are twisted away from the naphthalene ring. Two benzoyl groups at 1,8-positions of the naphthalene ring are situated in opposite directions, *anti* orientation. The dihedral angles between the benzene rings of benzoyl groups and the naphthalene ring system are 67.12 (5)° [C10—C1—C11—O1 torsion angle = -48.68 (15)°] and 85.15 (5)° [C10—C9—C18—O2 torsion angle = -59.99 (16)°], respectively. The dihedral angle between the best planes of the two benzene rings is 59.81 (6)°, which is distinctively larger than that of the homologous 1,8-dibenzoyl-2,7-dimethoxynaphthalene [12.18°]. The two benzoyloxy groups at 2,7-positions of naphthalene ring are also situated in opposite directions. The dihedral angles between the benzene rings of benzoyloxy groups and the naphthalene ring system are 71.47 (5)° and 76.41 (5)°, respectively. The phenyl rings and carbonyloxy moieties make almost coplanar [O4—C25—C26—C27 torsion angle = -5.7 (2)° and O6—C32—C33—C38 torsion angle = -8.86 (19)°].

In the crystal packing (Fig. 2), C–H···O interactions between the O1 oxygen atom of a carbonyl groups and the H15 hydrogen atoms of the C12–C17 phenyl ring are observed linking molecules into chains parallel to the *b* axis (Table 1). Further stabilization is provided by a C—H···π (Table 1) and by π–π stacking interactions [Cg1···Cg2^i^, 3.6441 (7) Å; Cg3···Cg3^ii^, 3.9197 (8) Å; Cg1, Cg2 and Cg3 are the centroids of the C1–C5/C10, C5–C10 and C26–C31 rings, respectively; symmetry codes: (i) 1-x, -y, 1-z; (ii) 2-x, -y, 1-z].

### Experimental

The title compound was prepared by reaction of 1,8-dibenzoyl-2,7-dihydroxynaphthalene (0.2 mmol, 73.68 mg), which was obtained *via* ethyl ether cleavage reaction of 1,8-dibenzoyl-2,7-diethoxynaphthalene, benzoyl chloride (0.4 mmol, 56.2 mg), and triethylamine (0.4 mmol, 40.5 mg) in dichloromethane (2.5 ml). After the reaction mixture was stirred at room temperature for 2 h, it was poured into water (30 ml) and the mixture was extracted with CHCl_3_ (10 ml × 3). The combined extracts were washed with brine. The organic layers thus obtained were dried over anhydrous MgSO_4_. The solvent was removed under reduced pressure to give the crude product, which was purified by recrystallization from dichloromethane. Colourless single crystals suitable for X-ray diffraction were obtained by repeated crystallization from dichloromethane (isolated yield 65%).

Spectroscopic data:^1^H NMR δ (300 MHz, CDCl_3_); 7.20–7.28 (8*H*, m), 7.38 (2*H*, t, *J*=13.5 Hz), 7.46 (2*H*, t, *J*=14.7 Hz), 7.54 (4*H*, d, *J*=7.5 Hz), 7.57 (2*H*, d, *J*=9.0 Hz), 7.74 (4*H*, d, *J*=7.8 Hz), 8.15 (2*H*, d, *J*=9.3 Hz) p.p.m.. ^13^C NMR δ (100 MHz, CDCl_3_); 122.20, 127.93, 128.25, 128.29, 129.94, 130.04, 130.87, 131.71, 133.26, 133.67, 138.27, 147.90, 163.99, 195.49 p.p.m.. IR (KBr); 1735 (OC═ O), 1662 (C═O), 1597 (Ar), 1507 (Ar) cm^-1^. M. p. = 524.9–525.9 K. Anal. Calcd for C38H24O6: C, 79.16; H, 4.20; Found: C, 79.74; H, 4.47.

### Refinement

All H atoms were found in a difference map and were subsequently refined as riding atoms, with C—H = 0.95 Å, and with *U_iso_*(H) = 1.2 *U*_eq_(C).

### Crystal data

**Table:**

C_38_H_24_O_6_	*F*(000) = 2400
*M**_r_* = 576.57	*D*_x_ = 1.351 Mg m^−^^3^
Orthorhombic, *P**b**c**a*	Cu *K*α radiation, λ = 1.54187 Å
Hall symbol: -P 2ac 2ab	Cell parameters from 84271 reflections
*a* = 18.0080 (3) Å	θ = 3.0–68.2°
*b* = 12.4307 (2) Å	µ = 0.74 mm^−^^1^
*c* = 25.3332 (4) Å	*T* = 193 K
*V* = 5670.89 (16) Å^3^	Block, colourless
*Z* = 8	0.40 × 0.40 × 0.10 mm

### Data collection

**Table:**

Rigaku R-AXIS RAPID diffractometer	5182 independent reflections
Radiation source: rotating anode	4687 reflections with *I* > 2σ(*I*)
Graphite monochromator	*R*_int_ = 0.023
Detector resolution: 10.000 pixels mm^-1^	θ_max_ = 68.2°, θ_min_ = 3.5°
ω scans	*h* = −21→21
Absorption correction: numerical (*NUMABS*; Higashi, 1999)	*k* = −14→14
*T*_min_ = 0.756, *T*_max_ = 0.930	*l* = −30→29
95847 measured reflections	

### Refinement

**Table:**

Refinement on *F*^2^	Secondary atom site location: difference Fourier map
Least-squares matrix: full	Hydrogen site location: inferred from neighbouring sites
*R*[*F*^2^ > 2σ(*F*^2^)] = 0.032	H-atom parameters constrained
*wR*(*F*^2^) = 0.084	*w* = 1/[σ^2^(*F*_o_^2^) + (0.0397*P*)^2^ + 1.6474*P*] where *P* = (*F*_o_^2^ + 2*F*_c_^2^)/3
*S* = 1.05	(Δ/σ)_max_ = 0.001
5182 reflections	Δρ_max_ = 0.18 e Å^−^^3^
398 parameters	Δρ_min_ = −0.16 e Å^−^^3^
0 restraints	Extinction correction: *SHELXL97* (Sheldrick, 2008), Fc^*^=kFc[1+0.001xFc^2^λ^3^/sin(2θ)]^-1/4^
Primary atom site location: structure-invariant direct methods	Extinction coefficient: 0.00092 (5)

### Special details

**Table:**

Geometry. All e.s.d.'s (except the e.s.d. in the dihedral angle between two l.s. planes) are estimated using the full covariance matrix. The cell e.s.d.'s are taken into account individually in the estimation of e.s.d.'s in distances, angles and torsion angles; correlations between e.s.d.'s in cell parameters are only used when they are defined by crystal symmetry. An approximate (isotropic) treatment of cell e.s.d.'s is used for estimating e.s.d.'s involving l.s. planes.
Refinement. Refinement of *F*^2^ against ALL reflections. The weighted *R*-factor *wR* and goodness of fit *S* are based on *F*^2^, conventional *R*-factors *R* are based on *F*, with *F* set to zero for negative *F*^2^. The threshold expression of *F*^2^ > σ(*F*^2^) is used only for calculating *R*-factors(gt) *etc*. and is not relevant to the choice of reflections for refinement. *R*-factors based on *F*^2^ are statistically about twice as large as those based on *F*, and *R*- factors based on ALL data will be even larger.

### Fractional atomic coordinates and isotropic or equivalent isotropic displacement parameters (Å^2^)

**Table:**

	*x*	*y*	*z*	*U*_iso_*/*U*_eq_	
O1	0.61898 (5)	0.15763 (7)	0.34605 (3)	0.0370 (2)	
O2	0.50065 (5)	−0.02769 (7)	0.35288 (4)	0.0409 (2)	
O3	0.75228 (4)	0.08534 (7)	0.45140 (3)	0.0338 (2)	
O4	0.76608 (5)	−0.06529 (8)	0.50018 (4)	0.0467 (2)	
O5	0.35421 (4)	0.12464 (6)	0.40337 (3)	0.03133 (19)	
O6	0.32765 (5)	0.30188 (7)	0.40896 (4)	0.0435 (2)	
C1	0.62268 (6)	0.09205 (9)	0.43251 (5)	0.0292 (2)	
C2	0.67885 (6)	0.09509 (9)	0.46926 (5)	0.0318 (3)	
C3	0.66720 (7)	0.11854 (10)	0.52281 (5)	0.0350 (3)	
H3	0.7077	0.1205	0.5468	0.042*	
C4	0.59685 (7)	0.13832 (10)	0.53959 (5)	0.0343 (3)	
H4	0.5883	0.1540	0.5758	0.041*	
C5	0.53608 (6)	0.13602 (9)	0.50417 (5)	0.0300 (2)	
C6	0.46371 (6)	0.15936 (9)	0.52283 (5)	0.0323 (3)	
H6	0.4564	0.1737	0.5593	0.039*	
C7	0.40452 (6)	0.16164 (9)	0.48963 (5)	0.0323 (3)	
H7	0.3564	0.1797	0.5022	0.039*	
C8	0.41618 (6)	0.13662 (9)	0.43633 (5)	0.0292 (2)	
C9	0.48431 (6)	0.11040 (9)	0.41559 (4)	0.0280 (2)	
C10	0.54791 (6)	0.11234 (9)	0.44988 (5)	0.0279 (2)	
C11	0.64142 (6)	0.08607 (9)	0.37478 (5)	0.0300 (3)	
C12	0.68743 (6)	−0.00277 (9)	0.35355 (5)	0.0315 (3)	
C13	0.69647 (7)	−0.09995 (10)	0.38020 (5)	0.0346 (3)	
H13	0.6726	−0.1109	0.4132	0.042*	
C14	0.73998 (7)	−0.18031 (11)	0.35889 (5)	0.0411 (3)	
H14	0.7463	−0.2462	0.3774	0.049*	
C15	0.77445 (8)	−0.16499 (11)	0.31054 (6)	0.0449 (3)	
H15	0.8046	−0.2201	0.2959	0.054*	
C16	0.76479 (8)	−0.06924 (12)	0.28359 (6)	0.0459 (3)	
H16	0.7880	−0.0591	0.2503	0.055*	
C17	0.72179 (7)	0.01137 (11)	0.30470 (5)	0.0395 (3)	
H17	0.7155	0.0769	0.2860	0.047*	
C18	0.48452 (6)	0.06636 (9)	0.35992 (5)	0.0306 (3)	
C19	0.46058 (6)	0.13750 (10)	0.31580 (5)	0.0337 (3)	
C20	0.42928 (7)	0.09152 (13)	0.27092 (5)	0.0463 (3)	
H20	0.4224	0.0158	0.2690	0.056*	
C21	0.40808 (9)	0.15631 (17)	0.22901 (6)	0.0618 (5)	
H21	0.3850	0.1252	0.1990	0.074*	
C22	0.42038 (8)	0.26547 (17)	0.23072 (6)	0.0623 (5)	
H22	0.4071	0.3092	0.2014	0.075*	
C23	0.45204 (8)	0.31167 (13)	0.27503 (6)	0.0531 (4)	
H23	0.4608	0.3870	0.2761	0.064*	
C24	0.47094 (7)	0.24812 (11)	0.31782 (5)	0.0408 (3)	
H24	0.4911	0.2803	0.3487	0.049*	
C25	0.79254 (7)	0.00012 (10)	0.47096 (5)	0.0346 (3)	
C26	0.86968 (6)	−0.00040 (10)	0.45085 (5)	0.0331 (3)	
C27	0.91357 (7)	−0.08882 (11)	0.46329 (6)	0.0406 (3)	
H27	0.8934	−0.1465	0.4833	0.049*	
C28	0.98659 (8)	−0.09286 (11)	0.44665 (6)	0.0438 (3)	
H28	1.0165	−0.1534	0.4552	0.053*	
C29	1.01600 (7)	−0.00930 (12)	0.41776 (6)	0.0454 (3)	
H29	1.0663	−0.0121	0.4065	0.054*	
C30	0.97251 (8)	0.07880 (12)	0.40502 (6)	0.0476 (3)	
H30	0.9930	0.1362	0.3850	0.057*	
C31	0.89918 (7)	0.08345 (11)	0.42147 (5)	0.0407 (3)	
H31	0.8693	0.1438	0.4127	0.049*	
C32	0.31154 (6)	0.21336 (10)	0.39400 (5)	0.0321 (3)	
C33	0.24452 (6)	0.18511 (10)	0.36282 (5)	0.0327 (3)	
C34	0.22400 (7)	0.07882 (11)	0.35420 (6)	0.0433 (3)	
H34	0.2530	0.0220	0.3684	0.052*	
C35	0.16118 (8)	0.05601 (13)	0.32482 (6)	0.0516 (4)	
H35	0.1475	−0.0167	0.3186	0.062*	
C36	0.11833 (7)	0.13831 (14)	0.30455 (6)	0.0500 (4)	
H36	0.0756	0.1222	0.2840	0.060*	
C37	0.13754 (7)	0.24401 (14)	0.31411 (6)	0.0495 (4)	
H37	0.1074	0.3006	0.3009	0.059*	
C38	0.20081 (7)	0.26746 (12)	0.34296 (5)	0.0419 (3)	
H38	0.2143	0.3403	0.3492	0.050*	

### Atomic displacement parameters (Å^2^)

**Table:**

	*U*^11^	*U*^22^	*U*^33^	*U*^12^	*U*^13^	*U*^23^
O1	0.0313 (4)	0.0409 (5)	0.0387 (5)	0.0039 (4)	0.0022 (4)	0.0094 (4)
O2	0.0448 (5)	0.0337 (5)	0.0442 (5)	0.0012 (4)	0.0010 (4)	−0.0063 (4)
O3	0.0234 (4)	0.0391 (5)	0.0387 (5)	0.0006 (3)	−0.0019 (3)	0.0049 (4)
O4	0.0345 (5)	0.0486 (5)	0.0571 (6)	−0.0002 (4)	0.0024 (4)	0.0164 (5)
O5	0.0238 (4)	0.0337 (4)	0.0366 (5)	0.0009 (3)	−0.0027 (3)	−0.0019 (3)
O6	0.0383 (5)	0.0340 (5)	0.0582 (6)	−0.0002 (4)	−0.0092 (4)	−0.0045 (4)
C1	0.0269 (6)	0.0265 (5)	0.0341 (6)	−0.0005 (4)	0.0007 (5)	0.0016 (5)
C2	0.0251 (6)	0.0323 (6)	0.0379 (7)	−0.0010 (4)	0.0001 (5)	0.0024 (5)
C3	0.0307 (6)	0.0393 (7)	0.0351 (7)	−0.0027 (5)	−0.0076 (5)	0.0002 (5)
C4	0.0353 (6)	0.0369 (6)	0.0306 (6)	−0.0026 (5)	−0.0017 (5)	−0.0002 (5)
C5	0.0304 (6)	0.0282 (6)	0.0314 (6)	−0.0022 (4)	−0.0003 (5)	0.0018 (5)
C6	0.0336 (6)	0.0339 (6)	0.0293 (6)	−0.0018 (5)	0.0036 (5)	−0.0005 (5)
C7	0.0273 (6)	0.0341 (6)	0.0355 (6)	−0.0011 (5)	0.0049 (5)	0.0007 (5)
C8	0.0251 (5)	0.0285 (6)	0.0341 (6)	−0.0025 (4)	−0.0020 (5)	0.0018 (5)
C9	0.0272 (6)	0.0263 (5)	0.0306 (6)	−0.0013 (4)	0.0002 (4)	0.0015 (4)
C10	0.0268 (6)	0.0253 (5)	0.0315 (6)	−0.0017 (4)	−0.0003 (5)	0.0018 (4)
C11	0.0220 (5)	0.0339 (6)	0.0342 (6)	−0.0036 (4)	−0.0012 (4)	0.0032 (5)
C12	0.0262 (5)	0.0357 (6)	0.0327 (6)	−0.0013 (5)	−0.0007 (5)	−0.0002 (5)
C13	0.0313 (6)	0.0391 (6)	0.0336 (6)	−0.0008 (5)	0.0017 (5)	0.0034 (5)
C14	0.0416 (7)	0.0362 (7)	0.0456 (8)	0.0045 (5)	0.0010 (6)	0.0053 (6)
C15	0.0448 (7)	0.0427 (7)	0.0473 (8)	0.0099 (6)	0.0079 (6)	−0.0024 (6)
C16	0.0506 (8)	0.0490 (8)	0.0381 (7)	0.0056 (6)	0.0135 (6)	0.0035 (6)
C17	0.0434 (7)	0.0385 (7)	0.0365 (7)	0.0025 (6)	0.0052 (5)	0.0059 (5)
C18	0.0226 (5)	0.0341 (6)	0.0350 (6)	−0.0024 (5)	0.0007 (4)	−0.0031 (5)
C19	0.0242 (5)	0.0474 (7)	0.0297 (6)	0.0033 (5)	0.0016 (5)	−0.0009 (5)
C20	0.0350 (7)	0.0671 (9)	0.0369 (7)	−0.0055 (6)	−0.0015 (5)	−0.0040 (6)
C21	0.0428 (8)	0.1077 (15)	0.0349 (8)	−0.0028 (9)	−0.0077 (6)	0.0057 (8)
C22	0.0429 (8)	0.0980 (14)	0.0459 (9)	0.0144 (9)	0.0010 (7)	0.0269 (9)
C23	0.0461 (8)	0.0589 (9)	0.0544 (9)	0.0169 (7)	0.0097 (7)	0.0183 (7)
C24	0.0376 (7)	0.0449 (7)	0.0399 (7)	0.0100 (6)	0.0025 (6)	0.0025 (6)
C25	0.0302 (6)	0.0365 (6)	0.0370 (7)	−0.0007 (5)	−0.0047 (5)	0.0026 (5)
C26	0.0290 (6)	0.0363 (6)	0.0341 (6)	−0.0001 (5)	−0.0040 (5)	−0.0017 (5)
C27	0.0387 (7)	0.0379 (7)	0.0451 (8)	0.0025 (5)	−0.0019 (6)	0.0017 (6)
C28	0.0380 (7)	0.0434 (7)	0.0498 (8)	0.0115 (6)	−0.0015 (6)	−0.0036 (6)
C29	0.0315 (7)	0.0573 (9)	0.0474 (8)	0.0065 (6)	0.0056 (6)	−0.0056 (7)
C30	0.0375 (7)	0.0528 (8)	0.0525 (8)	0.0019 (6)	0.0094 (6)	0.0090 (7)
C31	0.0340 (6)	0.0425 (7)	0.0457 (8)	0.0056 (5)	0.0009 (6)	0.0064 (6)
C32	0.0271 (6)	0.0347 (6)	0.0346 (6)	0.0008 (5)	0.0031 (5)	0.0003 (5)
C33	0.0254 (5)	0.0407 (6)	0.0321 (6)	0.0013 (5)	0.0021 (5)	−0.0013 (5)
C34	0.0341 (7)	0.0423 (7)	0.0536 (8)	0.0013 (6)	−0.0056 (6)	−0.0056 (6)
C35	0.0390 (7)	0.0568 (9)	0.0589 (9)	−0.0089 (6)	−0.0054 (7)	−0.0129 (7)
C36	0.0300 (6)	0.0806 (11)	0.0392 (7)	−0.0049 (7)	−0.0046 (6)	−0.0042 (7)
C37	0.0368 (7)	0.0674 (10)	0.0442 (8)	0.0065 (7)	−0.0061 (6)	0.0102 (7)
C38	0.0377 (7)	0.0447 (7)	0.0434 (8)	0.0018 (6)	−0.0033 (6)	0.0049 (6)

### Geometric parameters (Å, º)

**Table:**

O1—C11	1.2184 (14)	C18—C19	1.4890 (17)
O2—C18	1.2178 (15)	C19—C24	1.3886 (19)
O3—C25	1.3760 (14)	C19—C20	1.3917 (18)
O3—C2	1.4027 (14)	C20—C21	1.386 (2)
O4—C25	1.1984 (15)	C20—H20	0.9500
O5—C32	1.3649 (14)	C21—C22	1.375 (3)
O5—C8	1.4019 (13)	C21—H21	0.9500
O6—C32	1.1994 (15)	C22—C23	1.384 (2)
C1—C2	1.3751 (16)	C22—H22	0.9500
C1—C10	1.4388 (16)	C23—C24	1.3839 (19)
C1—C11	1.5027 (17)	C23—H23	0.9500
C2—C3	1.4035 (18)	C24—H24	0.9500
C3—C4	1.3588 (17)	C25—C26	1.4796 (17)
C3—H3	0.9500	C26—C31	1.3866 (18)
C4—C5	1.4155 (17)	C26—C27	1.3900 (17)
C4—H4	0.9500	C27—C28	1.3817 (19)
C5—C6	1.4163 (16)	C27—H27	0.9500
C5—C10	1.4226 (17)	C28—C29	1.377 (2)
C6—C7	1.3582 (17)	C28—H28	0.9500
C6—H6	0.9500	C29—C30	1.384 (2)
C7—C8	1.4013 (17)	C29—H29	0.9500
C7—H7	0.9500	C30—C31	1.3859 (19)
C8—C9	1.3738 (16)	C30—H30	0.9500
C9—C10	1.4376 (16)	C31—H31	0.9500
C9—C18	1.5129 (16)	C32—C33	1.4845 (17)
C11—C12	1.4817 (16)	C33—C38	1.3859 (18)
C12—C13	1.3935 (17)	C33—C34	1.3892 (18)
C12—C17	1.3946 (17)	C34—C35	1.3835 (19)
C13—C14	1.3797 (18)	C34—H34	0.9500
C13—H13	0.9500	C35—C36	1.381 (2)
C14—C15	1.386 (2)	C35—H35	0.9500
C14—H14	0.9500	C36—C37	1.380 (2)
C15—C16	1.383 (2)	C36—H36	0.9500
C15—H15	0.9500	C37—C38	1.3848 (19)
C16—C17	1.3747 (19)	C37—H37	0.9500
C16—H16	0.9500	C38—H38	0.9500
C17—H17	0.9500		
			
C25—O3—C2	116.55 (9)	C20—C19—C18	119.12 (12)
C32—O5—C8	117.77 (9)	C21—C20—C19	119.91 (15)
C2—C1—C10	118.47 (11)	C21—C20—H20	120.0
C2—C1—C11	119.67 (10)	C19—C20—H20	120.0
C10—C1—C11	121.09 (10)	C22—C21—C20	120.31 (15)
C1—C2—O3	118.21 (10)	C22—C21—H21	119.8
C1—C2—C3	123.37 (11)	C20—C21—H21	119.8
O3—C2—C3	118.07 (10)	C21—C22—C23	120.08 (15)
C4—C3—C2	118.65 (11)	C21—C22—H22	120.0
C4—C3—H3	120.7	C23—C22—H22	120.0
C2—C3—H3	120.7	C22—C23—C24	119.99 (16)
C3—C4—C5	121.25 (11)	C22—C23—H23	120.0
C3—C4—H4	119.4	C24—C23—H23	120.0
C5—C4—H4	119.4	C23—C24—C19	120.22 (14)
C4—C5—C6	119.70 (11)	C23—C24—H24	119.9
C4—C5—C10	120.09 (11)	C19—C24—H24	119.9
C6—C5—C10	120.20 (11)	O4—C25—O3	122.37 (11)
C7—C6—C5	121.31 (11)	O4—C25—C26	125.66 (11)
C7—C6—H6	119.3	O3—C25—C26	111.96 (10)
C5—C6—H6	119.3	C31—C26—C27	119.88 (12)
C6—C7—C8	118.32 (11)	C31—C26—C25	122.77 (11)
C6—C7—H7	120.8	C27—C26—C25	117.34 (11)
C8—C7—H7	120.8	C28—C27—C26	120.05 (13)
C9—C8—C7	123.71 (11)	C28—C27—H27	120.0
C9—C8—O5	117.25 (10)	C26—C27—H27	120.0
C7—C8—O5	118.57 (10)	C29—C28—C27	120.06 (12)
C8—C9—C10	118.45 (10)	C29—C28—H28	120.0
C8—C9—C18	116.39 (10)	C27—C28—H28	120.0
C10—C9—C18	124.56 (10)	C28—C29—C30	120.21 (12)
C5—C10—C9	117.92 (10)	C28—C29—H29	119.9
C5—C10—C1	118.17 (10)	C30—C29—H29	119.9
C9—C10—C1	123.91 (10)	C29—C30—C31	120.13 (13)
O1—C11—C12	120.84 (11)	C29—C30—H30	119.9
O1—C11—C1	118.09 (11)	C31—C30—H30	119.9
C12—C11—C1	121.05 (10)	C30—C31—C26	119.68 (12)
C13—C12—C17	119.17 (11)	C30—C31—H31	120.2
C13—C12—C11	122.38 (11)	C26—C31—H31	120.2
C17—C12—C11	118.45 (11)	O6—C32—O5	123.38 (11)
C14—C13—C12	120.30 (12)	O6—C32—C33	125.57 (11)
C14—C13—H13	119.9	O5—C32—C33	111.04 (10)
C12—C13—H13	119.9	C38—C33—C34	119.63 (12)
C13—C14—C15	120.03 (12)	C38—C33—C32	118.69 (11)
C13—C14—H14	120.0	C34—C33—C32	121.66 (11)
C15—C14—H14	120.0	C35—C34—C33	119.80 (13)
C16—C15—C14	119.88 (12)	C35—C34—H34	120.1
C16—C15—H15	120.1	C33—C34—H34	120.1
C14—C15—H15	120.1	C36—C35—C34	120.35 (14)
C17—C16—C15	120.40 (12)	C36—C35—H35	119.8
C17—C16—H16	119.8	C34—C35—H35	119.8
C15—C16—H16	119.8	C37—C36—C35	120.01 (13)
C16—C17—C12	120.22 (12)	C37—C36—H36	120.0
C16—C17—H17	119.9	C35—C36—H36	120.0
C12—C17—H17	119.9	C36—C37—C38	119.96 (14)
O2—C18—C19	121.95 (11)	C36—C37—H37	120.0
O2—C18—C9	118.98 (11)	C38—C37—H37	120.0
C19—C18—C9	118.96 (10)	C37—C38—C33	120.22 (13)
C24—C19—C20	119.42 (12)	C37—C38—H38	119.9
C24—C19—C18	121.43 (11)	C33—C38—H38	119.9
			
C10—C1—C2—O3	−174.10 (10)	C15—C16—C17—C12	−0.1 (2)
C11—C1—C2—O3	−4.11 (16)	C13—C12—C17—C16	−0.85 (19)
C10—C1—C2—C3	−0.96 (17)	C11—C12—C17—C16	−179.85 (12)
C11—C1—C2—C3	169.03 (11)	C8—C9—C18—O2	−111.05 (12)
C25—O3—C2—C1	−120.40 (12)	C10—C9—C18—O2	59.98 (15)
C25—O3—C2—C3	66.09 (14)	C8—C9—C18—C19	65.30 (14)
C1—C2—C3—C4	0.65 (19)	C10—C9—C18—C19	−123.68 (12)
O3—C2—C3—C4	173.80 (11)	O2—C18—C19—C24	−153.51 (12)
C2—C3—C4—C5	−0.25 (18)	C9—C18—C19—C24	30.26 (16)
C3—C4—C5—C6	−178.62 (11)	O2—C18—C19—C20	24.45 (17)
C3—C4—C5—C10	0.21 (18)	C9—C18—C19—C20	−151.78 (11)
C4—C5—C6—C7	177.65 (11)	C24—C19—C20—C21	−0.66 (19)
C10—C5—C6—C7	−1.18 (17)	C18—C19—C20—C21	−178.67 (12)
C5—C6—C7—C8	2.15 (17)	C19—C20—C21—C22	2.4 (2)
C6—C7—C8—C9	−0.37 (18)	C20—C21—C22—C23	−1.9 (2)
C6—C7—C8—O5	171.54 (10)	C21—C22—C23—C24	−0.4 (2)
C32—O5—C8—C9	−121.80 (11)	C22—C23—C24—C19	2.2 (2)
C32—O5—C8—C7	65.76 (13)	C20—C19—C24—C23	−1.64 (19)
C7—C8—C9—C10	−2.32 (17)	C18—C19—C24—C23	176.32 (11)
O5—C8—C9—C10	−174.33 (9)	C2—O3—C25—O4	2.77 (17)
C7—C8—C9—C18	169.28 (11)	C2—O3—C25—C26	−178.22 (10)
O5—C8—C9—C18	−2.73 (15)	O4—C25—C26—C31	−173.21 (13)
C4—C5—C10—C9	179.66 (10)	O3—C25—C26—C31	7.82 (17)
C6—C5—C10—C9	−1.52 (16)	O4—C25—C26—C27	5.6 (2)
C4—C5—C10—C1	−0.50 (16)	O3—C25—C26—C27	−173.33 (11)
C6—C5—C10—C1	178.32 (10)	C31—C26—C27—C28	0.3 (2)
C8—C9—C10—C5	3.17 (15)	C25—C26—C27—C28	−178.55 (12)
C18—C9—C10—C5	−167.69 (10)	C26—C27—C28—C29	0.0 (2)
C8—C9—C10—C1	−176.66 (11)	C27—C28—C29—C30	−0.3 (2)
C18—C9—C10—C1	12.48 (17)	C28—C29—C30—C31	0.2 (2)
C2—C1—C10—C5	0.86 (16)	C29—C30—C31—C26	0.2 (2)
C11—C1—C10—C5	−168.98 (10)	C27—C26—C31—C30	−0.4 (2)
C2—C1—C10—C9	−179.31 (10)	C25—C26—C31—C30	178.39 (13)
C11—C1—C10—C9	10.85 (17)	C8—O5—C32—O6	6.18 (17)
C2—C1—C11—O1	−121.04 (12)	C8—O5—C32—C33	−174.52 (9)
C10—C1—C11—O1	48.67 (15)	O6—C32—C33—C38	8.86 (19)
C2—C1—C11—C12	57.47 (15)	O5—C32—C33—C38	−170.42 (11)
C10—C1—C11—C12	−132.81 (11)	O6—C32—C33—C34	−169.72 (13)
O1—C11—C12—C13	−161.25 (11)	O5—C32—C33—C34	10.99 (16)
C1—C11—C12—C13	20.28 (17)	C38—C33—C34—C35	1.6 (2)
O1—C11—C12—C17	17.71 (17)	C32—C33—C34—C35	−179.88 (13)
C1—C11—C12—C17	−160.76 (11)	C33—C34—C35—C36	−0.7 (2)
C17—C12—C13—C14	1.15 (18)	C34—C35—C36—C37	−0.8 (2)
C11—C12—C13—C14	−179.89 (11)	C35—C36—C37—C38	1.6 (2)
C12—C13—C14—C15	−0.6 (2)	C36—C37—C38—C33	−0.7 (2)
C13—C14—C15—C16	−0.4 (2)	C34—C33—C38—C37	−0.8 (2)
C14—C15—C16—C17	0.7 (2)	C32—C33—C38—C37	−179.42 (12)

### Hydrogen-bond geometry (Å, º)

*Cg*2 is the centroid of the C5–C10 ring.

**Table:**

*D*—H···*A*	*D*—H	H···*A*	*D*···*A*	*D*—H···*A*
C15—H15···O1^i^	0.95	2.41	3.0584 (17)	125
C28—H28···*Cg*2^i^	0.95	2.65	3.4877 (14)	148

Symmetry code: (i) −*x*+3/2, *y*−1/2, *z*.
